# Human iPSC‐based models of Alzheimer’s disease

**DOI:** 10.1002/alz.087344

**Published:** 2025-01-09

**Authors:** Yanhong Shi

**Affiliations:** ^1^ Beckman Research Institute of City of Hope, Duarte, CA USA

## Abstract

**Background:**

Brain organoid models were generated from healthy control or Alzheimer’s disease patient iPSCs to facilitate our understanding of AD pathogenesis.

**Method:**

ApoE3 and ApoE4 iPSCs were developed into brain organoids using our recently developed brain organoid platform that allows prolonged culture of brain organoids. Human iPSCs were also differentiated into microglia, which were then co‐cultured with brain organoids. These neuroimmune organoids were used to uncover mechanisms underlying neurodegeneration.

**Result:**

We developed a long‐term brain organoid system that can undergo prolonged culture. Moreover, these organoids can support long‐term microglial co‐culture. Microglia can prevent neurons from degeneration in the long‐term brain organoids. ApoE4 microglia are less effective in reducing neuronal degeneration than ApoE3 microglia.

**Conclusion:**

The microglia‐containing long‐term brain organoid platform generated in this study provides a promising human cellular model for studying brain‐immune interactions in brain development and pathogenesis of neurological diseases such as AD.

